# Reply to ‘Response to “The opinion of French pulmonologists and palliative care physicians on non-invasive ventilation during palliative sedation at end of life: a nationwide survey’’ ’

**DOI:** 10.1186/s12904-023-01128-1

**Published:** 2023-03-06

**Authors:** V. Guastella, A. Greil, C. Lambert, A. Lautrette

**Affiliations:** 1 Palliative Care Unit, Montpied Hospital, University Hospital of Clermont-Ferrand, Clermont-Ferrand, France; 2 Pulmonology Unit, Montpied Hospital, University Hospital of Clermont-Ferrand, 54 Rue Montalembert, BP69, 63003 Cedex 1 Clermont-Ferrand, France; 3 Biostatistics Unit (DRCI), Montpied Hospital, University Hospital of Clermont-Ferrand, Clermont-Ferrand, France; 4 Medical Intensive Care, Montpied Hospital, University Hospital of Clermont-Ferrand, Clermont-Ferrand, France

**Keywords:** Palliative care, End of life, Non-invasive ventilation, Limitation of treatment

## Abstract

We read with interest the letter by Twycross and al on our article recently published in BMC Palliative Care. The authors suggest that the term palliative sedation has been used inappropriately and they consider that in the situation described the sedation was a procedural one rather than a continuous deep sedation. We strongly disagree with this point of view. In an end-of-life situation, the priorities are the patient’s comfort, pain and anxiety. This type of sedation does not have the characteristics of procedural sedation described in anaesthesia. The French Clayes Leonetti law makes it possible to clarify the intention of the sedation in end-of-life situations.

## Main text

We read with interest the letter by Twycross and al on our article recently published in BMC Palliative Care [1] reporting the results of a French nationwide survey about palliative care physicians’ and pulmonologists’ opinion on withdrawing or maintaining non-invasive ventilation (NIV) in patients with chronic respiratory failure during palliative sedation at end of life (EOL). We agree with many of the points made in this letter but strongly disagree with others and we thank the authors for the opportunity to clarify some of the elements of our paper.

The authors suggest that the term palliative sedation has been used inappropriately in relation to the outcomes of our work. We would like to clarify that throughout the manuscript we have only referred to the end-of-life situation of patients in chronic respiratory failure on NIV (a fact which was perfectly clear to the doctors who participated in the study). The authors of this letter consider that in the situation described the sedation was a procedural one rather than a continuous deep sedation. We strongly disagree with this point of view. The procedural sedation described in the reference mentioned in Twycross’s letter [2] is done in the context of an anaesthetic procedure which is described as: "The practice of procedural sedation is the administration of one or more pharmacological agents to facilitate a diagnostic or therapeutic procedure while targeting a state during which airway patency, spontaneous respiration, protective airway reflexes, and hemodynamic stability are preserved, while alleviating anxiety and pain.” In the end-of-life situation, airway protection and preservation of hemodynamic stability are not the priorities over patient comfort, pain and anxiety. Furthermore, for NIV in chronic respiratory failure patients outside the EOL situation, no procedural sedation is performed. In an end-of-life situation, the sedation proposed does not have the characteristics of procedural sedation described in anaesthesia. The sedation we spoke about in our paper aims to eliminate the discomfort that leads to significant anxiety and which considerably degrades the quality of death. In chronic respiratory failure patients on NIV, the end-of-life is regularly associated with significant dyspnoea and a sensation of asphyxia as described in the manuscript. These symptoms are dependent on the respiratory status, which in fact cannot improve, and on the patient's state of consciousness. Sedation is therefore intended to influence the only parameter that we can control in the end-of-life situation in this type of patient. Thus the sedation used will aim to achieve the loss of consciousness to make the perception of a situation experienced as unbearable disappear because of the asphyxia before death. We agree with the position of the authors of letter and the international community that the sedation is intended to provide the most effective relief and not to cause the patient's rapid death.

The Clayes Leonetti law [3] allows us and asks us to carry out this type of sedation in all end-of-life circumstances and not just “certain” as the authors of the letter stated.

Following the implementation of the Clayes Leonetti law in France in 2016, the French palliative care society developed the SEDAPALL score on sedative practices in the palliative context [4] to clarify the intention of the sedation by seeking the patient's consent, including duration and depth of sedation.

Finally, with these clarifications, we fully agree with the authors of the letter and as we suggested in our paper, there is a need for close collaboration between palliative care specialists and pulmonologists or other specialists. The other specialists need to learn the tools and culture of palliative care and the palliative care specialists, the specificities of the patients' diseases in order to improve the quality of EOL and the death of patients.

## Data Availability

Not applicable.

## Abbreviations

EOL: End of life
NIV: Non invasive ventilation
